# Dog Neuter, Yes or No? A Summary of the Motivations, Benefits, and Harms, with Special Emphasis on the Behavioral Aspect

**DOI:** 10.3390/ani15071063

**Published:** 2025-04-06

**Authors:** Ana Arroube, Alfredo F. Pereira

**Affiliations:** Mediterranean Institute for Agriculture, Environment and Development, Universidade de Évora, Apartado 94, 7006-554 Évora, Portugal

**Keywords:** neutering, castration, gonadectomy, behavior, dogs, review, anxiety, fear, disease risk

## Abstract

The decision to neuter pet dogs remains a significant and ongoing debate from ethical, animal health, and animal welfare perspectives. Millions of dogs worldwide are considered integral family members, and their behavior, as well as the bond they form with their owners, play a key role in the decision to keep them as pets. When behavioral problems arise, neutering is often viewed as a potential solution. However, by removing the source of sexual hormones, neutering creates the potential for both beneficial and harmful effects on a dog’s health and behavior. It is crucial for society to rely on professionals to provide informed advice about whether and when the procedure should be performed on a pet, taking into account the unique risks and benefits of each individual case. Instead, the conclusions drawn from existing research—both in terms of behavior and health aspects—remain inconclusive and sometimes conflicting. Studies have reported higher risks for developing reproductive, urinary, metabolic, and musculoskeletal disorders in neutered dogs. Additionally, some studies suggest increases in stress, fear, anxiety, and even certain types of aggression following neutering. The limitations of current research, the subjectivity of pet owners’ evaluations of their dogs’ behaviors, the influence of various demographic factors, and the complex relationships between sex hormones and other hormones or neurotransmitters all contribute to the challenges in this field of study. Despite these complexities, they also offer promising avenues for future research. This review aims to provide a comprehensive overview of the various effects of neutering on dogs, with a particular focus on behavioral outcomes.

## Simple Summary

Neutering pet dogs is a decision that, at some point, most owners will consider and, in some countries, is even mandatory by law or highly recommended as part of responsible pet ownership. Therefore, it is crucial to understand the long-term effects of this common procedure on an animal’s physical and mental health. However, the existing literature does not allow us to draw a single conclusion, as both advantages and disadvantages of the procedure have been described. Recently, more attention has been placed on the negative aspects, particularly concerning behavioral outcomes. Studies indicate that dogs may be at a heightened risk of developing fear, anxiety, and panic responses. Conversely, neutering, especially in males, is still recommended as a means of mitigating certain undesirable behaviors. Sexual hormones are interconnected with other hormones and neurotransmitters, such as cortisol, oxytocin, dopamine, and serotonin. Additionally, breed and sex also influence the effects of neutering. Demographic factors play a role, as well. More research is necessary to fully understand the implications of neutering and its intricate relationships. In the meantime, decisions regarding this procedure should be made on an individual basis, and alternative techniques for gonad removal may be explored.

## 1. Introduction

There are millions of dogs worldwide. In Europe alone, an estimated 70 million pet dogs existed in 2023 [1], while the United States (U.S.) leads the world with an estimated 90 million pet dogs [2]. Among the main reasons for acquiring a dog, studies consistently point to companionship, whether for the owner or a household member, as the most common [3,4,5,6,7,8]. Pets provide both physical and mental benefits to their owners [9,10,11,12,13]. In the U.S., a 2023 Pew Research Center survey found that 97% of pet owners consider their pets to be part of the family [14], and some even regard them as children [15].

Despite the strong bond between dogs and their owners, various factors can disrupt this connection, leading to relinquishment. In the U.S., the Shelter Animal Counter’s mid-year 2024 report noted that 22,000 dogs were surrendered to shelters, representing 31% of total intakes [16]. Research over time has examined the role that behavioral incompatibilities in dogs play in this problem. In 1996, Houpt et al. discussed aggression as one of the primary reasons for the disruption of the human–animal bond [17]. Several other studies have identified behavioral issues, such as biting, aggression toward people, destructive behaviors, and house soiling, as key factors in relinquishment [18,19,20]. Recent retrospective research also highlights behavior problems as a leading cause for dog relinquishment, with aggression topping the list at 28% of cases [21]. However, it is important to note that Patronek et al. (2021) reviewed the existing literature and concluded that the prevalence of significant behavioral incompatibilities may be overestimated in many studies [22].

Neutering is often seen as a solution to some unwanted behaviors and a means of preventing relinquishment, but it can also exacerbate or introduce new behavioral issues. Despite its potential drawbacks, neutering is a common routine surgery for many pet dogs. According to various studies, the prevalence of neutering varies across regions. In the United States, approximately 64% of dogs are neutered [23], while in the United Kingdom, the rate is 54% [24], and, in Ireland, it is 47% [25]. However, there is significant global disparity regarding this procedure. In the U.S., approximately 32 states require that all dogs or cats adopted from shelters be sterilized, with violations punishable through civil and criminal penalties [26]. Neutering is commonly performed in shelters on young animals as early as 6–8 weeks of age as a method of contraception to help address the pet overpopulation issue. In the U.K., most U.S. states, and some European countries, neutering is considered part of “responsible ownership” and is routinely carried out. In contrast, some German and Scandinavian countries (including Denmark, Finland, Iceland, Norway, and Sweden) have much more restrictive policies on neutering. For example, in Germany, the Animal Welfare Act prohibits neutering, except for health reasons or to control populations. In Norway, neutering is illegal unless strictly necessary [27].

Other reviews have explored the consequences of neutering [28,29,30,31,32,33,34], and the findings drawn from the literature are often inconclusive, with sometimes conflicting results across the three primary approaches to neutering—behavioral, health, and population control. Regarding health issues, research over the past few decades has pointed to an association between neutering and an increased risk for various diseases, including long-term reproductive, urinary, metabolic, and musculoskeletal adverse effects. However, the results for many of these conditions can be inconsistent [30]. For instance, one of the common reasons for advocating for neutering in females is the decreased risk of developing mammary tumors. However, a systematic review concluded that the evidence linking neutering to a reduced risk of mammary tumors—and the effect of the age at which it is performed—was weak [35]. In males, neutering reduces the risk of canine prostatic hyperplasia but may also increase the risk of other prostatic diseases, such as canine prostatic neoplasia [36]. The mechanisms through which the absence of gonads influences the increased risk for certain diseases are not fully understood. Some studies suggest that the continuous elevation of luteinizing hormone (LH) at supraphysiological levels after gonadectomy may play a role, as LH receptors are present in various normal tissues. This relationship warrants further investigation in future research [37,38].

Regarding behavioral concerns, which is the primary focus of this review, conflicting information persists in the literature [34]. First, it is important to understand the motivations behind neutering dogs, particularly when owners have behavioral expectations. These expectations can influence the decision to neuter or not. Some owners may hesitate to neuter their dogs based on beliefs that the procedure alters the dog’s personality or diminishes its “maleness” [39,40,41]. Others argue that neutering promotes inactivity [42] and could potentially have negative effects on the dog’s behavior [43]. On the other hand, neutering can also be viewed as a means to better control unwanted behaviors, such as fighting or straying [25]. A study in Poland identified undesirable behavior as the main reason for neutering male dogs, with hyperactivity and roaming accounting for 8% each and aggression and marking for 5% each [44]. Similarly, 58% of Dutch owners of neutered dogs reported that correcting unwanted behavior was a reason for castration, with aggression being the most common behavior (reported by 50% of the owners) [45]. Da Costa et al. (2021) found that neutering was often performed to prevent or correct aggressive behavior, particularly in male dogs [46]. In a study of English Springer Spaniels, 17% of males were castrated due to aggression toward humans and 10% for aggression toward other dogs [47]. It is evident that many owners view neutering as a way to address unwanted behaviors, particularly in male dogs.

As noted earlier, research findings are inconsistent. Early studies suggested that intact dogs displayed more problematic behaviors, and neutering was thought to improve certain behaviors, such as roaming, mounting, urinary marking, and intermale aggression [48,49,50,51]. These early conclusions likely contributed to the widespread belief that neutering is a reliable solution for unwanted behaviors. However, more recent studies have raised concerns about the potential for neutering to increase fear, anxiety, and aggression-related behaviors in both male and female dogs. Neutered dogs have been reported to exhibit more fear, nervousness, panic, social withdrawal, and even heightened aggression [44,52,53,54,55,56,57]. Therefore, caution must be exercised when considering neutering as a solution for behavioral issues.

The conflicting information regarding both the health and behavioral consequences of neutering makes it difficult to make decisions with confidence. Studying behavior can be particularly complex due to the many confounding factors involved. These factors include intrinsic elements like sex, breed, age, weight, and size of the dog, as well as extrinsic influences, such as the dog–owner lifestyle, the owner’s personality, demographics, culture, and societal values. All of these variables contribute to the development of a dog’s personality and behavior. Given this complexity, it is easy to see how challenging it can be to directly correlate neutering with behavior changes observed after the procedure.

Research is evolving, and some studies have started to investigate more specific factors. For instance, certain studies have explored the effect of the age at which castration occurs and the dog’s lifetime exposure to gonadal hormones [56,57,58,59,60], with more recent research also focusing on the influence of breeds [54,61]. Additionally, the hormonal shifts caused by the suppression of gonadal hormones undoubtedly play a role, although the full extent of these changes is not yet entirely understood. Testosterone, estradiol, oxytocin, serotonin, dopamine, cortisol, and progesterone are all affected by neutering, resulting in imbalances and shifts in their dynamics. Given the wide variability in study results, it is advisable to evaluate each case on an individual basis. It is also important to consider alternatives to definitive gonadectomy, such as other surgical options or chemical sterilization.

## 2. The Behavior Consequences of Dog Neutering

### 2.1. The 1970–1990s

A chronological review of the history of research in canine behavioral genetics shows that studies in canine behavior date to the early 1900s [62]. The research from Scott and Fuller in 1965 was a landmark in understanding many aspects of dog behavior and influenced and inspired later works. Based on twenty years of research, these two psychology professors gathered information on the role of genetic and dog behavior, studying many aspects, including dog sexual behavior [63]. Research on the impact of neutering on dog behavior has evolved significantly over the years. Hopkins et al. in 1976 concluded that male behavioral patterns were reduced by castration in adulthood. Roaming, for instance, was reduced in 90% of dogs. Fighting with other males, urine marking in the house, and mounting of other dogs or people were also reduced [48]. In a 1983 study, Borchelt investigated agonistic behavior in dogs, categorized into barking, biting, and growling. The study revealed that male dogs demonstrated significantly higher rates of aggression than females (67.4% vs. 32.6%). Within the male group, intact dogs were considerably more aggressive than castrated ones (86% vs. 14%). Conversely, among females, spayed individuals exhibited higher levels of aggression than intact females (68% vs. 32%) [64]. While the limitations of the study are addressed later in this paper, we contend that the findings should be interpreted with caution. The study is retrospective in nature, which introduces the potential for bias, as dog owners may have been predisposed to neuter their pets with the expectation of behavioral improvements, particularly in male dogs. Furthermore, the ratio of intact to castrated males, as well as the ratio of intact to spayed females, within the study’s population is unknown, which may affect the generalizability of the results. Similarly, Wright and Nesselrote in 1987 described that significantly more males and neutered females were referred for aggression and reactivity. The study was one of the first to intend to classify behavior problems in dogs and relate them with age, breed, sex, and reproductive status [65]. In the 1990s, several studies emerged addressing neutering and its effects on behavior. One study noted that changes in behavior following castration were more prominent in males than females. Generally, aggression decreased in dogs with a history of aggressive behavior, although some spayed females exhibited an increase in aggression. Specifically, this was noted in females who had displayed aggressive tendencies prior to neutering [66]. Most studies up to this point had focused primarily on males. In 1990, a study on females was published, supporting earlier findings that spayed females were more likely to display aggression, particularly dominant aggression toward family members, if they had exhibited such behavior in puppyhood [67]. In 1991, Salmeri et al. examined the age at castration. Despite the limitations discussed by the authors, they concluded that neutered dogs were generally more active than intact dogs, with males castrated at 7 weeks being the most excitable [68].

Research consistently shows that behaviors like roaming, fighting, mounting, and urinary marking in males tend to decrease following neutering. As noted by Wright and Nesselrote in 1987, more intact males were referred for behavior problems, as these behaviors were considered undesirable by owners. Early studies primarily focused on males, and changes in behavior after neutering were consistently observed in this group. The reduction in these behaviors in males, which are often mediated by testosterone, was discussed in several studies. Testosterone plays a significant role in dimorphic behaviors in dogs, and if these behaviors are hormonally driven, they may decrease or even disappear after castration. However, it is important not to overlook the influence of learning, experience, and personality.

Sexual mounting and copulatory behaviors, for instance, are directly influenced by testosterone in the medial preoptic area of the anterior hypothalamus. Thus, the removal of testosterone is expected to reduce these behaviors [34]. Mounting behavior itself is complex and can manifest in various contexts, such as during an exciting event or as a sign of stress, and it can occur in both sexes. Likewise, urinary marking and intermale aggression are behaviors that may happen in different contexts, and they can also be influenced by prior experiences and learning. While urinary marking is often distressing to owners, it is not solely controlled by hormones. It does not depend on testosterone’s effects on the preoptic–anterior hypothalamus [34], and context and individual experience play significant roles, as well. A recent study by Kaufmann et al. in 2017 found no significant differences in behaviors like mounting, overmarking, and urination with leg raised between neutered and intact male dogs. These are some of the most common reasons for neutering males, suggesting that castration may not be effective for these reasons [53].

Early studies have limitations, as previously mentioned in other reviews. A significant flaw was the lack of control groups of sexually intact dogs, making it challenging to draw meaningful conclusions. Control groups are crucial for distinguishing outcomes caused by the neutering procedure from those resulting from other factors, such as changes in the household or a new activity, like attending training classes. Additionally, many studies were retrospective, relying on historical data, owner memories, and subjective descriptions of behavior [65]. Retrospective studies are more prone to inaccuracies, and relying on owner descriptions introduces the risk of bias. Furthermore, the distinction between non-pathological and pathological unwanted behaviors was not always made, meaning that an owner may describe a problem behavior in a similar way in which they would describe the same behavior motivated by an underlying pathology.

Sampling bias was another issue, as certain types of owners or dogs were more likely to be included, such as those with known aggressive behavior problems, like in Borchelt’s 1983 study, or those selected based on a specific outcome. The sample size in many studies was small, which makes it harder to generalize the results. Furthermore, dogs were often not studied in terms of breed, age, individuality, or age at castration. The categorization of behavior in some studies was also inconsistent, making it difficult to compare results between different research efforts. For example, aggression might have been broadly classified without considering underlying causes like pain or fear, which could lead to misinterpretation of the behavior.

Despite these limitations, the studies from the 1970s, 1980s, and 1990s were crucial in bringing attention to the potential behavioral changes associated with neutering. These early studies laid the groundwork for more recent research that has benefited from improved study designs and methodology.

### 2.2. From the 2000s Onwards

Below, we list some of the key studies on the topic, organized chronologically from the 2000s to the present (Table 1). For each study, we provide a summary of the year, sample categorization, design, and results. It is important to note that the results and conclusions are reviewed from a behavioral perspective, as this is the primary focus of the paper.

The Evolution of Research: Over time, research in this field has led to significant improvements in several aspects, although many studies still require cautious interpretation. Early studies from the 1970s to the 1990s primarily focused on male dogs, their behavioral patterns, and the effects of castration on these behaviors. However, more recent studies have begun to reveal potentially concerning behavioral changes in both sexes. While behaviors once considered problematic by owners, such as roaming, mounting, urinary marking, and intermale aggression, were consistently shown to decline in earlier studies, they did not entirely disappear. Instead, they became less emphasized or were no longer the central findings of the research. In contrast, newer studies increasingly report behaviors related to fear, phobias, panic, anxiety, and certain types of aggression, which may actually increase following castration. The growing consistency and frequency of these findings can likely be attributed to improvements in study designs and sample sizes and the refinement of criteria and behavioral classifications. These advancements have paved the way for a more comprehensive exploration of these behaviors.

A notable flaw in earlier research was the absence of control groups, which made it difficult to draw meaningful conclusions. The lack of a control group limits the ability to compare results with a baseline, making it hard to determine the effects of the castration procedure in isolation. Randomly assigning dogs to different groups addresses this issue and helps ensure that confounding factors do not influence the results, thus providing a more rigorous methodology compared to retrospective studies. Additionally, as previously mentioned, many early studies were retrospective in nature. However, since the 2000s, more prospective studies have been conducted, offering stronger evidence. A common methodology in these studies has been the use of questionnaires and surveys. The sample sizes in these studies ranged from 400 to 13,700 participants, highlighting the ability of questionnaires to collect large data sets quickly and efficiently.

Furthermore, the development of questionnaires has improved over time. Early studies relied on open-ended questions and owner descriptions, often followed up with phone interviews. This approach was subject to varying interpretations by owners and inconsistencies in the questions asked, depending on the researcher. However, more recent studies have benefited from more standardized and carefully designed questionnaires, ensuring more reliable and consistent data collection. Prior to the 2000s, validated tools for assessing dog behavior were unavailable. However, starting in the 2000s, systematic questionnaires began to emerge to better assess and understand dog behavior, including how it relates to various factors, such as reproductive status. One well-known example is the “Canine Behavioral Assessment and Research Questionnaire” (C-BARQ) [56,57,58,70,74,83]. This questionnaire allows owners to answer a series of questions about their dog’s behavior in different situations, helping to identify behavioral patterns and potential issues. Despite their advantages, studies using questionnaires and surveys have their limitations. Many studies have relied on convenience samples, typically recruited from locations where dog owners are easily accessible, such as pet shops, veterinary clinics, hospitals, and dog-related websites. While convenient, these samples are prone to research bias. Several steps can be taken to mitigate this bias, but it remains a concern. For instance, owners who frequent veterinary clinics and hospitals and are willing to participate in surveys may possess certain characteristics, such as being more responsible pet owners, being better informed, and having moderate socioeconomic status [56,57]. Another potential confounding factor is the origin of the dogs themselves. Dogs from shelters, for example, are more likely to exhibit behavioral problems and be neutered, especially when compared to purebred dogs or those acquired as puppies.

Classification of Dog Behavior: Another flaw in early studies was the inconsistent classification of behaviors. Although this remains an issue in some studies, the emergence of research on canine personality has likely contributed to a more standardized classification of dog behavior, allowing for better comparison across studies. Moreover, recent studies have incorporated more demographic factors, focusing on how both the dog’s and the owner’s characteristics relate to the animal’s personality traits. However, as discussed in the study by Salonen et al. (2021), the traits that constitute a dog’s personality are not straightforward [84]. Different studies have defined varying traits, leading to some inconsistency. For instance, the Dog Personality Questionnaire (DPQ) defines traits, such as fearfulness, aggression toward people, aggression towards animals, activity/excitability, and responsiveness to training. The Monash Canine Personality Questionnaire (MCPQ-R) includes traits like extraversion, motivation, training focus, amicability, and neuroticism [84]. Similarly, Turcsán et al. (2011) identified four key personality traits: sociability, trainability, calmness, and boldness [85]. Kubinyi et al. (2009) also categorized 17 out of 24 traits into four factors: trainability, boldness, calmness, and sociability [73]. Several studies mentioned earlier have linked personality traits to castration, among other variables [54,55,70,74,75,79,85].

Demographic and Environmental Factors: In the past, studies focused on relating behavior to variables like sex, gender, and reproductive status and also breed [86]. However, more recent research in dog behavior and health has highlighted that numerous factors influence dog behavior. These factors can range from aggressive behavior to susceptibility to joint disorders. Beyond the dog’s intrinsic characteristics, such as sex, age, and breed, factors related to the owner and the living environment also play a crucial role. For example, housing conditions, the number of household members (including children), the presence of other dogs, and the dog’s routines and activities (e.g., training classes, daycare attendance, dog parks) all influence future behavior. These factors can also change over the course of a dog’s life and after events like neutering.

Challenges in Establishing Causality: Given the vast number of confounding factors, establishing clear causation between behavior and neutering remains challenging. Although more studies since the 2000s have incorporated demographic variables into their designs, further research is needed to better isolate and understand the specific influence of neutering on behavior. Well-defined study designs are essential for obtaining reliable results. Another important factor to consider is the dog’s genetic background. Recent studies have explored the relationship between neutering and specific breeds [52,54,61].

## 3. Hormonal Changes Associated with Neutering

Increased anxiety, panic, and fear reactions have been reported in numerous studies as potential consequences of neutering [44,52,53,54,55,56,57,58,59,60,73,74,75]. Of particular note, sound phobias have been highlighted, as several studies report an increase in this phobia following neutering [44,52,59]. One study even identified a correlation between the age at gonadectomy and the development of noise phobia, indicating that dogs neutered at 5.5 months of age are more likely to exhibit this behavior [28]. The observed increases in stress and anxiety, predominantly described in males, as well as aggression (which can be driven by underlying anxiety and fear), have been discussed in relation to alterations in levels of testosterone, oxytocin, dopamine, serotonin, cortisol, and progesterone.

Testosterone: It is well-documented in various species that the presence of testosterone reduces fear and anxiety-like behaviors. In male mice, for example, exogenous testosterone reduced aversion to cat odor [87], and its absence or deficiency facilitated an enhanced fear response [88]. Similarly, heifers treated with testosterone exhibited reduced fear and reactivity to novel stimuli [89]. In humans, although testosterone influences a wide range of psychological traits, low levels have been associated with various manifestations of anxiety, ranging from generalized fear to phobic anxiety and full-blown panic disorders [90]. Therefore, it is plausible that low levels of testosterone, or its absence, may also affect fear and anxiety responses in dogs. Additionally, some studies suggest that cortisol competes with sex steroid hormones for binding sites [55]. Fear-related aggression is modulated by stress hormones, such as cortisol, with testosterone acting as an antagonist. Consequently, in the absence of testosterone following neutering, increased fear and insecurity may be expected [53].

Aggression in males and its association with testosterone have been widely documented. It is commonly believed that castration reduces aggression, particularly in males. This belief was initially influenced by early studies. In 1983, Borchelt reported that intact males were significantly more aggressive than neutered ones, with an 86% versus 14% difference in aggression rates. However, he also noted that for non-aggressive cases, the figures were 78% for intact males and 22% for castrated males, resulting in an imbalanced comparison [64]. A few years later, Wright and Nesselrote observed that 48% of dogs referred for problem behavior management, with aggression being a primary concern, were intact males, while only 12% were neutered males [65]. The limitations of these studies, which have been discussed earlier, include their retrospective design and biased sampling of dogs already exhibiting problematic behaviors. This could lead to an overestimation of aggression in intact dogs compared to neutered ones.

In 1990, Heinderbenger and Unshelm reported that neutering generally improved aggressive behavior in both male and female dogs [66]. Some studies have also shown a decrease in intra-specific aggression in males following neutering [44,48]. However, these findings are not consistent across research, as other studies have reported an increase in dog–dog aggression [66] or no changes at all [58]. The complexity of this topic suggests that several factors beyond study limitations (such as retrospective design, biased samples, limited demographic factors, and inadequate control of confounding variables) may contribute to varying results. One such factor is the breed. While Kolkmeyer et al. (2021) found that neutered dogs, regardless of breed, were more anxious, stressed, and aggressive [54], a more recent study in 2024 [61] suggested breed-dependent effects on aggression and stress-related responses. Wójcik and Powierzá (2021) found an increase in certain types of aggressive behaviors, particularly toward other dogs, which were breed-specific. They observed that undesirable behaviors, such as aggression toward humans and other dogs/animals, were most prevalent in breeds like Akitas, Siberian Huskies, and Samoyeds, with males being more affected [91].

Other factors, such as the presence of other dogs, can also influence aggression. In a 2018 study, Jacobs et al. investigated resource guarding aggression in dogs in the presence of other dogs and concluded that neutered males were more likely to exhibit this behavior [78]. These findings highlight the importance of examining different types of aggression individually, rather than treating aggression as a monolithic behavior and linking it solely to neutering.

Aggression toward owners and family members was reduced in some studies [51], while in others it was found to increase [47,59,76,77]. In 2024, Kolkmeyer et al. reported that in the Husky clade, aggression in general was primarily observed in intact dogs, whereas in the Bulldog clade, a higher number of neutered males exhibited aggressive behaviors. Interestingly, aggression toward household members was only observed in the Husky clade [61]. Similarly, aggression toward strangers was found to decrease in some studies [51], while in others it was found to increase [56,58,71].

In a 2001 review, Simpson discussed the role of testosterone in aggression, emphasizing that testosterone is just one of many factors influencing aggressive behavior and highlighting the importance of experience and the environment [92]. Therefore, it is overly simplistic to consider neutering as a solution for preventing or correcting aggression-related issues. Ayrosa et al. (2023) argued that “most of the literature has focused on biased views of breeding, behavior profiles, and aggression itself… but factors such as skull morphology, size, weight, caretaker relationships, and culture should be further incorporated into research for a deeper understanding of dog aggression as a social communicative behavior” [93]. Breed [94], as well as physical characteristics, such as height, body weight, and skull shape [95], have already been linked to aggression. Additionally, aspects related to the owner should also be considered. For example, owners may not always recognize changes in their dog’s behavior or may lack awareness of stress signals [96]. This highlights the complexity of establishing a reliable relationship between neutering and aggression, given the multitude of contributing factors. More research is needed to account for these variables in a comprehensive manner.

In female dogs, the ovaries are a source of testosterone production [97]. Following neutering and the removal of the gonads, there is likely a decrease in circulating testosterone levels, as demonstrated in the study by Hydbring-Sandberg et al. (2021), which examined the short-term effects of neutering on testosterone and other hormones in bitches [98]. However, in women and prepubertal children, the adrenal glands serve as an important source of androgens and androgen precursors [99]. Therefore, a compensatory effect by the adrenal glands in the absence of the ovaries may occur, although this hypothesis requires further investigation in dogs.

Oxytocin: Oxytocin (OT) is another critical hormone involved in regulating a variety of behaviors. This neuropeptide, produced in the paraventricular and supraoptic nuclei of the hypothalamus, exerts both central and peripheral effects, functioning as a neuromodulator in the latter case [100]. In humans, evidence suggests that oxytocin reduces fear responses to social stimuli [101], and it is known to have anxiolytic, anti-stress, and generally prosocial effects [102]. OT is believed to interact with the amygdala, a region of the brain responsible for regulating social behavior and emotions, such as fear and anxiety. Research indicates that oxytocin reduces the activation of the amygdala, thereby modulating fear and anxiety processing [102].

While the positive effects of oxytocin are well-documented, the role and dynamics of this hormone are complex, both in humans and non-human species. Although both sexes have oxytocin receptors, OT seems to have a more pronounced effect in females, as its synthesis and receptor activity are heavily regulated by estrogens. Conversely, a similar neuropeptide, vasopressin, plays a more significant role in males, though both sexes have receptors for both neuropeptides, and these molecules can bind to each other’s receptors [103]. Another area of complexity regarding oxytocin is its involvement in aggression. As noted by Kirsch et al. (2005), the contribution of oxytocin to aggressive behavior is intricate and may depend on the specific context of social interactions. Studies in primates and rodents have supported this hypothesis [101].

In dogs, it can be assumed that oxytocin plays a role in promoting positive social behavior. Research has shown that OT influences social interactions, such as how dogs behave toward humans, particularly their friendliness toward strangers [104], as well as their gaze behavior and the strength of human–dog bonding [105]. Additionally, oxytocin has been shown to enhance a dog’s play motivation [106]. Because oxytocin synthesis and receptor activity are estrogen-regulated, particular attention should be given to the relationship between oxytocin and neutering in female dogs. As previously mentioned, some research has indicated an increase in aggression in bitches following neutering. A recent study suggested that if oxytocin receptors are activated by sex hormones, and those hormones are no longer present after neutering, this could lead to a decrease in oxytocin levels, potentially contributing to the behavioral changes observed in neutered dogs [55].

Oxytocin also functions as an antagonist to cortisol [79]. The reduction in estrogen levels in females after neutering could disrupt oxytocin levels and impair amygdala-mediated processing of fear and anxiety, potentially explaining the observed increase in aggression in neutered females. This aggression may be triggered by underlying fear or anxiety. Research has consistently linked neutering in females with increased aggression. As early as 1983, Borchelt reported that spayed females were much more likely to be aggressive than intact females [64], and Wright and Nesselrote (1987) noted that neutered females and intact males were the most commonly referred dogs for aggression and stimulus reactivity behavior problems [65]. These findings raised concerns about the behavioral effects of castration in females. More recent studies focusing specifically on female dogs have largely supported these concerns, with neutered females displaying greater reactivity, fear, aggression, and nervousness [57,69,71,75,77,79]. However, many of these studies were breed-specific, limiting their broader applicability.

For example, Balogh et al. (2018) found that gonadectomized female Labrador Retrievers were more fearful in specific situations. They observed a relationship between increased aggression toward an approaching dog and the timing of gonadectomy, with females neutered after puberty showing the strongest effects [77]. In contrast, Moxon et al. (2022), working with crossbred female Labradors and Golden Retrievers, concluded that gonadectomy, regardless of whether it occurred before or after puberty, had no significant impact on future behavior, including aggression [82]. Kim et al. (2005, 2006) conducted prospective studies with German Shepherd females and found an increase in reactivity and vocalization following neutering [69,71]. Another study reported that gonadectomized females exhibited greater anxiety and nervousness, along with increased aggression toward humans, including household members [78]. In 2019, Starling et al. linked the percentage of a dog’s lifetime exposure to gonadal hormones (PLGH) with various behaviors, finding that reduced PLGH was associated with higher incidences of fear, anxiety, aggression, and excitability [57].

Serotonin and Dopamine: In 2021, Hydbring-Sandberg et al. studied the concentrations of serotonin, cortisol, testosterone, and progesterone in the urine of female dogs shortly after ovariohysterectomy. They observed a decrease in the urinary serotonin/creatinine ratio one week after the procedure, but, by four weeks post-surgery, the serotonin levels tended to be higher than before surgery, an effect that could not be fully explained. Additionally, a positive correlation with cortisol and progesterone levels was noted following ovariohysterectomy. Urinary testosterone levels significantly decreased after the procedure [98]. Similarly, in 2024, Guvenc-Bayram et al. found decreased serotonin levels in both sexes after gonadectomy, measured 7 and 14 days post-surgery. Notably, they observed a significant difference in serotonin levels between males and females, with females consistently showing higher levels both pre- and post-neutering [107]. These findings align with evidence suggesting that females may benefit more from a protective effect of estrogens on serotonin pathways than males [108,109]. However, further measurements over time would be beneficial to better understand these dynamics.

Serotonin modulates a wide range of behaviors, including aggression, impulsivity, food selection, sexual behaviors, and mood [110]. In humans, depression and anxiety are often linked to low serotonergic neurotransmission, and the amygdala, which regulates emotional responses, such as fear and anxiety, contains serotonin receptors [111]. Estrogens are known to enhance serotonergic activity, and with neutering leading to a reduction in sex steroid levels, behavioral changes, such as increased fear and anxiety, are expected, particularly in females.

The dopaminergic and serotonergic systems are functionally and anatomically interconnected, and research suggests an inverse relationship between these two systems in the context of aggression [112]. Guvenc-Bayram et al. observed an increase in dopamine levels shortly after neutering, followed by a decline below baseline levels by the 14th day post-surgery. The authors suggested that this decline might indicate a disruption in dopamine pathways and an adaptation of the reward system. Such changes may not only affect metabolism—one of the study’s main goals—but also influence behavior [107]. Dopamine plays a critical role in learned behavior, with dopamine-deficient animals being unable to perform basic functions, such as searching for food, obtaining rewards, or avoiding punishment. Dopamine is also closely tied to motivation, with lower levels typically associated with reduced motivation. As dopamine levels increase, motivation tends to rise to a certain threshold [113].

It could be speculated that lower dopamine levels in neutered animals may contribute to reduced trainability and a diminished motivation to repeat rewarded behaviors. However, this remains speculative, as research on the effect of neutering on trainability presents conflicting results. Some studies have suggested that neutered females and intact males are more trainable [73], while others found intact females to be more trainable [79]. In contrast, research involving German Shepherds reported that both intact males and females were more trainable than their neutered counterparts [80]. Other studies found no relationship between neutering and trainability in females of any breed, although positive effects on trainability were observed in male Shepherd dogs [70].

Cortisol: Cortisol is a key hormone that helps both humans and animals manage stress. In dogs, cortisol levels can serve as an indicator of their well-being and their response to environmental factors. Dogs that experience stable routines, positive interactions, environmental enrichment, and regular socialization tend to exhibit lower stress levels [114]. Elevated cortisol levels have been observed in dogs displaying various types of aggression compared to controls [115]. In a study by Sandri et al. (2010), lower cortisol levels were reported in neutered dogs of both sexes [116]. This finding warrants further investigation to better understand the underlying mechanisms.

Progesterone: Some studies have reported that progesterone levels are lower in neutered dogs of both sexes [98,117]. In the study by Hydbring-Sandberg et al. (2021), a positive correlation between serotonin and progesterone was observed, with progesterone being produced by the adrenal glands following gonadectomy [117]. In humans, progesterone has been shown to influence behavior and mental health through its receptors in the brain. These receptors are widespread in areas of the brain that regulate behavior, such as the amygdala [118]. The fluctuations of progesterone in neutered dogs and its potential effects on behavioral responses require further investigation.

## 4. Alternatives to Gonadal Removal

Due to the ongoing uncertainties surrounding the risks and benefits of neutering in dogs, an alternative and potentially safer approach may involve a surgical procedure that preserves the gonads, thereby maintaining their physiological effects on tissue maturation and overall physiology. Kutzler (2020) outlines two techniques for gonad-sparing sterilization in dogs: ovariohysterectomy and vasectomy [119]. This approach preserves normal levels of sexual hormones, meaning that behaviors associated with sexual dimorphism, such as roaming, urinary marking, mounting, and male-to-male aggression, are unlikely to be diminished. Therefore, this method is not suitable if the goal is to reduce such behaviors. However, it may be indicated for preventing breeding or controlling populations. One downside of this procedure is the stress and pain typically associated with any form of invasive surgery.

An alternative to surgical neutering is the use of non-surgical methods, such as gonadotropin-releasing hormone (GnRH) agonists administered via slow-release implants, such as Deslorelin. While some side effects have been observed in female dogs, Deslorelin is considered a safe and effective drug for inducing temporary infertility in male dogs, male cats, male ferrets, and prepubertal female dogs [120]. During initial administration, Deslorelin stimulates GnRH receptors, leading to an increase in circulating sex hormones, which may result in undesired physiological and behavioral effects. This is referred to as the “flare-up” (FU) effect. This phase is followed by desensitization of the GnRH receptors. To mitigate the FU effect, Cyproterone acetate, a synthetic progestogen, can be used in conjunction with Deslorelin, offering an alternative to surgical neutering [121]. This method avoids the risks associated with general anesthesia and the stress and pain of surgery. Notably, it allows for safe and reversible observation of the effects of gonadal hormone suppression in animals, particularly when the goal of neutering is to alter natural behavior. This approach may be suitable for behavioral modification and breeding control, although in the latter case, permanent alternatives may be more appropriate.

## 5. Conclusions

The development of evidence-based guidelines for the optimal timing of canine neutering to mitigate significant health and behavioral issues is critical. Existing evidence indicates that gonadectomy induces hormonal alterations that appear to contribute to various adverse effects on both canine health and behavior. The physiological implications of neutering warrant further investigation not only at the level of individual hormones and neurotransmitters but also in terms of the broader, dynamic interactions between them. The complexities introduced by breed-specific genetic factors and environmental influences make it challenging to accurately assess the full impact of neutering on behavioral outcomes. In the interim, alternative approaches, such as gonad-sparing sterilization, should be explored as potential strategies to maintain hormonal equilibrium.

## Figures and Tables

**Table 1:** Relevant studies of behavioral consequences of dog neutering, in chronological order, from 2000 onwards.

Research Study	Year	Sample Categorization	Study Design	Results
Spain and Houpt [59]	2004	1842 dogs adopted from shelter. Both sexes.	Retrospective cohort study	Males and females neutered at an early age had an increase in noise phobias and sexual behaviors; separation anxiety, escaping behaviors, inappropriate elimination when frightened, and relinquishment for any reason were decreased. Aggression toward family members was more frequent among male dogs gonadectomized at an early age.
Reisner et al. [47]	2005	1053 adult English Springer Spaniels. Both sexes.	Prevalence survey	Sex and neuter status were two variables related to aggressive behavior toward familiar people. Neutered males and females were more aggressive than intact ones in the majority of contexts of owner-directed aggression. Neutered males and females were significantly more likely to have bitten. Neutered females were also more aggressive toward unfamiliar adults who approached.
Kim et al. [69]	2005	60 German Sheperd females.	Prospective study	There was a reported increase in territorial aggression in the ovariohysterectomy group. Neutered females showed more posture and aggressive expressions than the control. The acoustic feature changed, with a higher rate of barking call in the ovariohysterectomy group.
Serpell and Hsu [70]	2005	1593 dogs. Both sexes.	Cross- sectional study	Dogs were assessed for trainability. Neutering was not associated with any differences in trainability in female dogs in any breed but was associated with positive effects on trainability in male Shetland sheepdogs.
Kim et al. [71]	2006	14 German Sheperd females.	Prospective study	Dogs in the ovariohysterectomy group showed an increase in reactivity. Neutered females showed a more offensive posture and aggressive expressions than the control.
Bennett and Rohlf [72]	2007	413 participants. Both sexes.	Cross- sectional survey	When associating the frequency of potentially problematic behaviors with demographic variables, involvement in dog training activities, and participation in other dog–human interactions, neutered dogs of both sexes were found to be more nervous or timid than intact ones. Neutered dogs were also rated as less anxious and engaged in fewer destructive behaviors.
Kubinyi et al. [73]	2009	10,519 dogs. Both sexes.	Cross- sectional study	In both sexes (more significant in males), dogs with the lowest mean calmness were neutered and less than 2.5 years old. Neutered females and intact males were considered more trainable then their counterparts. Neutered dogs who spent less than 3 h with their owner daily were reported to be bolder and less bold if more than 3 h.
Starling et al. [74]‌	2013	1054 dogs. Both sexes.	Cross- sectional study	Male dogs were bolder than female dogs, and intact dogs were bolder than neutered ones. The study also suggests that neutering alters a dog’s willingness to engage in social behaviors and/or their desire to avoid novel or potentially frightening nonsocial objects.
Zink et al. [60]	2014	2505 Vizlas. Both sexes.	Retrospective cohort study	Neutered Vizlas are more at risk of developing several behavioral problems (for example, fear and aggression) than intact ones, but this was entirely influenced by the age at which dogs were gonadectomized. Neutering at ≤6 months poses a greater risk.
Tiira et al. [75]	2016	3284 dogs. Both sexes.	Cross- sectional survey	Neutered females presented more avoidance behaviors, intact males more urinating/defecating and destroying behavior, and neutered dogs of both sexes more panting and pacing and less vocalization toward loud noises. Males and neutered dogs had a more excited/active type of behavior in new situations.
Bálint et al. [76]	2017	93 dogs. Both sexes	Experimental study	A relationship was found between dogs’ age and reproductive status. Dogs between 2 and 6 years of age showed a difference based on their reproductive status; intact dogs were significantly less “obedient” than the neutered ones. A significant interaction between behavior type based on the “roll over” test and the reproductive status of the dog was found. The intact “resistant” dogs showed the lowest “aggressive towards owner” scores, but neutered “resistant” dogs had the highest scores for aggression.
Kaufmann et al.‌ [53]	2017	18 intact male dogs and 16 neutered male dogs, 104 male dogs for questionnaires, 54 case studies on male dogs.	Observational study	Intact dogs are socially more active toward neutered ones (smelling and licking the genital area, chin rest, tooth chatter, and molesting). As for the questionnaires, intact dogs seem to be bolder, more sociable, and less trainable. As for the case studies, the biggest difference was fearful behavior. Neutered males seemed more anxious during walks and more aggressive toward other dogs. Neutering may have a negative influence on the behavior of male dogs.
Balogh et al. [77]‌	2018	58 female Labrador Retrievers.	Experimental study	The owners of neutered females described their dogs as having more intense or more frequent fear reactions in response to loud noises, strange or unfamiliar objects on or near the sidewalk, or if unknown jumping, barking, or growling dogs were approaching them (this last one occurred mostly in females neutered after puberty).
Farhoody et al. [58]‌	2018	13,500 dogs. Both sexes.	Cross- sectional study	There was a minimal increase in aggression toward strangers for all neutered dogs compared with intact dogs, which was mostly seen in the group of dogs gonadectomized at 7–12 months of age.
Jacobs et al. [78]	2018	3589 dogs Both sexes.	Cross- sectional study	Among other characteristics, neutered males were more likely to be resource guarding aggressive compared to dogs of other sexes and neuter statuses.
McGreevy et al. [56]	2018	6235 neutered male dogs.	Cross- sectional survey	Some forms of aggression, mainly related to fear, were significantly and positively associated with lower age at neutering and percentage of lifetime exposure to gonadal hormones (PLGH). Other behaviors, like indoor marking, increased with PLGH.
Lorenz et al. [79]	2019	180 female dogs.	Observational study	Intact female dogs were significantly calmer and more trainable and sociable toward other dogs. Neutered females tended to be less bold than intact ones, more anxious and nervous, trembled more often, and were more aggressive toward humans in general and specific objects.
Starling et al. [57]	2019	8981 neutered female dogs.	Cross- sectional survey	Female dogs with less exposure to their natural gonadal hormones (decreased PLGH) showed greater incidence of several fear/anxiety, aggressive, and excitable behaviors than intact female dogs in several contexts.
Fattah and Hamid [80]	2020	120 German Sheperd dogs. Both sexes.	Experimental design	Males were significantly more trainable than females. As for sexual status, both intact males and females were significantly more trainable than the neutered ones.
Hakanen et al. [52]‌	2020	13,700 dogs. Both sexes.	Cross- sectional survey	Neutered dogs showed more non-social fears. They were more likely to show fear of fireworks and thunder than intact dogs. Intact males were less fearful in novel situations, and intact females were less fearful when compared to neutered females.
Palestrini et al. [81]	2021	96 dogs. Both sexes.	Cross-sectional study	Neutered dogs were reported to show less mounting behavior, pulling on the leash, and roaming behaviors. Marking behavior did not vary across time for both groups of dogs. A tendency to reduce owner-directed aggression was observed at the interview 9 months after surgery for male dogs, while no change was observed for male controls.
Kolkmeyer et al. [54]	2021	230 dogs. Both sexes.	Cross- sectional study	Intact males were more open-minded and less fearful than neutered dogs. Neutered dogs showed significantly greater panic reactions compared to intact ones and higher values for aggressive behavior.
Moxon et al.‌ [82]	2022	155 and 151 Labrador and Golden Retrievers crossbred. Females.	Prospective cohort study	The results suggest that for Labrador and Golden Retriever crossbred bitches, neutering before or after puberty has little to no effect on future behavior.
Kriese et al. [44]	2022	396 male dogs.	Online survey	Neutering reduced aggressive behaviors toward dogs and other animals. An increase in the number of dogs that were fearful of unfamiliar dogs/humans was reported, as well as sound phobias. The surgery greatly decreased incidences of roaming, mounting, and urine marking as well as the dog’s overall activity.
Kolkmeyer et al. [55]	2024	230 mixed-breed dogs. Male dogs.	Correlational study	Neutered dogs appeared more stressed than intact ones, especially related to human noises and other reasons like car rides or unfamiliar environments. Neutered dogs differed significantly from intact dogs in terms of general aggression and emotional stability; they were much less social, less trainable, emotionally calm, and extroverted than intact dogs.
Kolkmeyer et al. [61]	2024	136 dogs from Huskie and Bulldog clades. Male dogs.	Cross- sectional study	An increase in aggression toward humans is described for neutered males in both clades. An increase in aggression in walks and toward dogs in mostly seen in the Bulldog clade. As for stress-related responses, there is a significant difference in stress due to other dogs and noises and more panic reactions, stress, and uncertainty in neutered dogs.

## Data Availability

No new data were created in this paper, which serves as a comprehensive review of existing knowledge.

## References

[1] Statista. https://www.statista.com/statistics/515010/pet-population-european-union-eu-by-animal/.

[2] World Population Review. https://worldpopulationreview.com/country-rankings/dog-population-by-country.

[3] Holland K.E. (2019). Acquiring a Pet Dog: A Review of Factors Affecting the Decision-Making of Prospective Dog Owners. Animals.

[4] Holland K.E., Mead R., Casey R.A., Upjohn M.M., Christley R.M. (2022). Why Do People Want Dogs? A Mixed-Methods Study of Motivations for Dog Acquisition in the United Kingdom. Front. Vet. Sci..

[5] Jagoe A., Serpell J. (1996). Owner characteristics and interactions and the prevalence of canine behaviour problems. Appl. Anim. Behav. Sci..

[6] Ortega-Pacheco A., Bolio-Gonzalez M.E., Colin-Flores R.F., Sauri-Arceo C.H., Gutierrez-Blanco E., Jimenez-Coello M., Linde Forsberg C. (2006). Evaluation of a Burdizzo castrator for neutering of dogs. Reprod. Domest. Anim..

[7] Packer R.M.A., Brand C.L., Belshaw Z., Pegram C.L., Stevens K.B., O’Neill D.G. (2021). Pandemic Puppies: Characterising Motivations and Behaviours of UK Owners Who Purchased Puppies during the 2020 COVID-19 Pandemic. Animals.

[8] Powell L., Chia D., McGreevy P., Podberscek A.L., Edwards K.M., Neilly B., Guastella A.J., Lee V., Stamatakis E. (2018). Ex-pectations for dog ownership: Perceived physical, mental and psychosocial health consequences among prospective adopters. PLoS ONE.

[9] Bauman A.E., Russell S.J., Furber S.E., Dobson A.J. (2001). The epidemiology of dog walking: An unmet need for human and canine health. Med. J. Aust..

[10] Levine G.N., Allen K., Braun L.T., Christian H.E., Friedmann E., Taubert K.A., Thomas S.A., Wells D.L., Lange R.A. (2013). Pet Ownership and Cardiovascular Risk. Circulation.

[11] Serpell J. (1991). Beneficial Effects of Pet Ownership on Some Aspects of Human Health and Behaviour. J. R. Soc. Med..

[12] Thorpe R.J., Simonsick E.M., Brach J.S., Ayonayon H., Satterfield S., Harris T.B., Garcia M., Kritchevsky S.B. (2006). Dog Ownership, Walking Behavior, and Maintained Mobility in Late Life. J. Am. Geriatr. Soc..

[13] Teo J.T., Johnstone S.J., Römer S.S., Thomas S.J. (2022). Psychophysiological mechanisms underlying the potential health benefits of human-dog interactions: A systematic literature review. Int. J. Psychophysiol..

[14] Pew Research Center. https://www.pewresearch.org/short-reads/2023/07/07/about-half-us-of-pet-owners-say-their-pets-are-as-much-a-part-of-their-family-as-a-human-member/.

[15] Greenebaum J. (2004). It’s a Dog’s Life: Elevating Status from Pet to “Fur Baby” at Yappy Hour. Soc. Anim..

[16] Shelter Animal County. https://www.shelteranimalscount.org/2024-mid-year-report.

[17] Houpt K.A., Honig S.U., Reisner I.R. (1996). Breaking the human-companion animal bond. J. Am. Vet. Med. Assoc..

[18] Diesel G., Brodbelt D., Pfeiffer D.U. (2010). Characteristics of Relinquished Dogs and Their Owners at 14 Rehoming Centers in the United Kingdom. J. Appl. Anim. Welf. Sci..

[19] Miller D.D., Staats S.R., Partlo C., Rada K. (1996). Factors associated with the decision to surrender a pet to an animal shelter. J. Am. Vet. Med. Assoc..

[20] Salman M.D., New J.G., Scarlett J.M., Kass P.H., Ruch-Gallie R., Hetts S. (1998). Human and Animal Factors Related to Re-linquishment of Dogs and Cats in 12 Selected Animal Shelters in the United States. J. Appl. Anim. Welf. Sci..

[21] Kisley M.A., Chung E.J., Levitt H. (2024). Investigating the Reasons behind Companion Animal Relinquishment: A Systematic Content Analysis of Shelter Records for Cats and Dogs, 2018–2023. Animals.

[22] Patronek G.J., Bradley J., Arps E. (2021). Saving Normal: A new look at behavioral incompatibilities and dog relinquishment to shelters. J. Vet. Behav..

[23] Trevejo R., Yang M., Lund E.M. (2011). Epidemiology of surgical castration of dogs and cats in the United States. J. Am. Vet. Med. Assoc..

[24] Diesel G., Brodbelt D., Laurence C. (2010). Survey of veterinary practice policies and opinions on neutering dogs. Vet. Rec..

[25] Downes M.J., Devitt C., Downes M.T., More S.J. (2015). Neutering of cats and dogs in Ireland; pet owner self reported perceptions of enabling and disabling factors in the decision to neuter. Peer J..

[26] Michigan State University. https://www.animallaw.info/intro/state-spay-and-neuter-laws.

[27] Fossati P. (2022). Spay/neuter laws as a debated approach to stabilizing the populations of dogs and cats: An overview of the European legal framework and remarks. J. Appl. Anim. Welf. Sci..

[28] Houlihan K.E. (2017). A literature review on the welfare implications of gonadectomy of dogs. J. Am. Vet. Med. Assoc..

[29] Root Kustritz M.V. (2012). Effects of surgical sterilization on canine and feline health and on society. Reprod. Domest. Anim..

[30] Root Kustritz M.V., Slater M.R., Weedon G.R., Bushby P.A. (2017). Determining optimal age for gonadectomy in the dog: A critical review of the literature to guide decision making. Clin. Theriogenology.

[31] Mckenzie B. (2010). Evaluating the benefits and risks of neutering dogs and cats. CAB Rev. Perspect. Agric. Vet. Sci. Nutr. Nat. Resour..

[32] Reichler I.M. (2009). Gonadectomy in cats and dogs: A review of risks and benefits. Reprod. Domest. Anim..

[33] Salmeri K.R., Olson P.N., Bloomberg M.S. (1991). Elective gonadectomy in dogs: A review. J. Am. Vet. Med. Assoc..

[34] Urfer S.R., Kaeberlein M. (2019). Desexing Dogs: A Review of the Current Literature. Animals.

[35] Beauvais W., Cardwell J.M., Brodbelt D.C. (2012). The effect of neutering on the risk of mammary tumours in dogs--a systematic review. J. Small Anim. Pract..

[36] Schrank M., Romagnoli S. (2020). Prostatic Neoplasia in the Intact and Castrated Dog: How Dangerous is Castration?. Animals.

[37] Kutzler M.A. (2020). Possible Relationship between Long-Term Adverse Health Effects of Gonad-Removing Surgical Sterilization and Luteinizing Hormone in Dogs. Animals.

[38] Zwida K., Kutzler M.A. (2016). Non-reproductive long-term health complications of gonad removal in dogs as well as possible causal relationships with post-gonadectomy elevated luteinizing hormone (LH) concentrations. J. Etiol. Anim. Health.

[39] Cocia R.I., Rusu A.S. (2010). Attitudes of Romanian Pet Caretakers towards Sterilization of Their Animals: Gender Conflict Over Male, but Not Female, Companion Animals. Anthrozoös.

[40] McKay S.A., Farnworth M.J., Waran N.K. (2009). Current Attitudes Toward, and Incidence of, Sterilization of Cats and Dogs by Caregivers (Owners) in Auckland, New Zealand. J. Appl. Anim. Welf. Sci..

[41] Egwu G.O., Mamza S.A., Mshelia G.D., UBA M.A. (2016). Preponderances of dog owners to neutering of companion animals: A survey of dog owners in Maiduguri metropolis, Borno State, Nigeria. Med. Res. Chron..

[42] Wongsaengchan C., McKeegan D. (2019). The Views of the UK Public Towards Routine Neutering of Dogs and Cats. Animals.

[43] Ong D., Barboza de Moura Santos M.J., Thomsen D.A., Feakes A.M. (2017). Australian owners’ attitudes and experiences of having a dog neutered. Aust. Vet. Pract..

[44] Kriese M., Kuźniewska E., Gugołek A., Strychalski J. (2022). Reasons for and Behavioral Consequences of Male Dog Castration—A Questionnaire Study in Poland. Animals.

[45] Roulaux P.E.M., van Herwijnen I.R., Beerda B. (2020). Self-reports of Dutch dog owners on received professional advice, their opinions on castration and behavioural reasons for castrating male dogs. PLoS ONE.

[46] Da Costa R.E.P., Kinsman R.H., Owczarczak-Garstecka S.C., Casey R.A., Tasker S., Knowles T.G., Woodward J.L., Lord M.S., Murray J.K. (2022). Age of sexual maturity and factors associated with neutering dogs in the UK and the Republic of Ireland. Vet. Rec..

[47] Reisner I.R., Houpt K.A., Shofer F.S. (2005). National survey of owner-directed aggression in English Springer Spaniels. J. Am. Vet. Med. Assoc..

[48] Hopkins S.G., Schubert T.A., Hart B.L. (1976). Castration of adult male dogs: Effects on roaming, aggression, urine marking, and mounting. J. Am. Vet. Med. Assoc..

[49] Maarschalkerweerd R.J., Endenburg N., Kirpensteijn J., Knol B.W. (1997). Influence of orchiectomy on canine behaviour. Vet. Rec..

[50] Knol B.W., Egberink-Alink S.T. (1989). Treatment of problem behaviour in dogs and cats by castration and progestagen admin-istration: A review. Vet. Q..

[51] Neilson J.C., Eckstein R.A., Hart B.L. (1997). Effects of castration on problem behaviors in male dogs with reference to age and duration of behavior. J. Am. Vet. Med. Assoc..

[52] Hakanen E., Mikkola S., Salonen M., Puurunen J., Sulkama S., Araujo C., Lohi H. (2020). Active and social life is associated with lower non-social fearfulness in pet dogs. Sci. Rep..

[53] Kaufmann C.A., Forndran S., Stauber C., Woerner K., Gansloßer U. (2017). The Social Behaviour of Neutered Male Dogs Compared to Intact Dogs (Canis Lupus Familiaris): Video Analyses, Questionnaires and Case Studies. Vet. Med. Open J..

[54] Kolkmeyer C.A., Schmitz J., Gansloßer U. (2021). Behavioural correlates of Neutering Male Dogs—A question of Breed. J. Veter. Sci. Med..

[55] Kolkmeyer C.A., Zambrano Cardona A.M., Gansloßer U. (2024). Personality Unleashed: Surveying Correlation of Neuter Status and Social Behaviour in Mixed-Breed Male Dogs across Weight Classes. Animals.

[56] McGreevy P.D., Wilson B., Starling M.J., Serpell J.A. (2018). Behavioural risks in male dogs with minimal lifetime exposure to gonadal hormones may complicate population-control benefits of desexing. PLoS ONE.

[57] Starling M., Fawcett A., Wilson B., Serpell J., McGreevy P. (2019). Behavioural risks in female dogs with minimal lifetime exposure to gonadal hormones. PLoS ONE.

[58] Farhoody P., Mallawaarachchi I., Tarwater P.M., Serpell J.A., Duffy D.L., Zink C. (2018). Aggression toward Familiar People, Strangers, and Conspecifics in Gonadectomized and Intact Dogs. Front. Vet. Sci..

[59] Spain C.V., Scarlett J.M., Houpt K.A. (2004). Long-term risks and benefits of early-age gonadectomy in dogs. J. Am. Vet. Med. Assoc..

[60] Zink M.C., Farhoody P., Elser S.E., Ruffini L.D., Gibbons T.A., Rieger R.H. (2014). Evaluation of the risk and age of onset of cancer and behavioral disorders in gonadectomized Vizslas. J. Am. Vet. Med. Assoc..

[61] Kolkmeyer C.A., Baum J., Warlich-Zach N., Gansloßer U. (2024). From “Husky” to “Bulldog”- behavioural correlates between castration and breed groups in the domestic dog (*Canis lupus familiaris*). BMC Vet. Res..

[62] Mackenzie S.A., Oltenacu E.A., Houpt K.A. (1986). Canine behavioral genetics—A review. Appl. Anim. Behav. Sci..

[63] Scott J.P., Fuller J.L. (1965). Genetics and the Social Behavior of the Dog.

[64] Borchelt P.L. (1983). Aggressive behavior of dogs kept as companion animals: Classification and influence of sex, reproductive status and breed. Appl.Anim. Ethol..

[65] Wright J.C., Nesselrote M.S. (1987). Classification of behavior problems in dogs: Distributions of age, breed, sex and reproductive status. Appl. Anim. Behav. Sci..

[66] Heidenberger E., Unshelm J. (1990). Changes in the behavior of dogs after castration. Tierarztl. Prax..

[67] O’Farrell V., Peachey E. (2008). Behavioural effects of ovariohysterectomy on hitches. J. Small Anim. Pract..

[68] Salmeri K.R., Bloomberg M.S., Scruggs S.L., Shille V. (1991). Gonadectomy in immature dogs: Effects on skeletal, physical, and behavioral development. J. Am. Vet. Med. Assoc..

[69] Kim H.H., Yeon S.C., Houpt K.A., Lee H.C., Chang H.H., Lee H.J. (2005). Acoustic feature of barks of ovariohysterectomized and intact German Shepherd bitches. J. Vet. Med. Sci..

[70] Serpell J.A., Hsu Y.A. (2005). Effects of breed, sex, and neuter status on trainability in dogs. Anthrozoös.

[71] Kim H.H., Yeon S.C., Houpt K.A., Lee H.C., Chang H.H., Lee H.J. (2006). Effects of ovariohysterectomy on reactivity in German Shepherd dogs. Vet. J..

[72] Bennett P.C., Rohlf V.I. (2007). Owner-companion dog interactions: Relationships between demographic variables, potentially problematic behaviours, training engagement and shared activities. Appl. Anim. Behav. Sci..

[73] Kubinyi E., Turcsán B., Miklósi Á. (2009). Dog and owner demographic characteristics and dog personality trait associations. Behav. Process..

[74] Starling M.J., Branson N., Thomson P.C., McGreevy P.D. (2013). Age, sex and reproductive status affect boldness in dogs. Vet. J..

[75] Tiira K., Sulkama S., Lohi H. (2016). Prevalence, comorbidity, and behavioral variation in canine anxiety. J. Vet. Behav..

[76] Balint A., Rieger G., Miklosi A., Pongracz P. (2017). Assessment of owner-directed aggressive behavioural tendencies of dogs in situations of possession and manipulation. R. Soc. Open Sci..

[77] Balogh O., Borruat N., Andrea Meier A., Hartnack S., Reichler I.M. (2018). The influence of spaying and its timing relative to the onset of puberty on urinary and general behaviour in Labrador Retrievers. Reprod. Domest. Anim..

[78] Jacobs J.A., Coe J.B., Pearl D.L., Widowski T.M., Niel L. (2018). Factors associated with canine resource guarding behaviour in the presence of dogs: A cross-sectional survey of dog owners. Prev. Vet. Med..

[79] Lorenz K.P., Kolkmeyer C.A., Ganslosser U. (2019). Comparison of the Social Behaviour of Intact and Neutered Female Domestic Dogs (*Canis lupus familiaris*): Questionnaires and Case Studies. Dairy Vet. Sci. J..

[80] Fattah A., Hamid S. (2020). Influence of gender, neuter status, and training method on police dog narcotics olfaction performance, behavior and welfare. J. Adv. Vet. and Anim. Res..

[81] Palestrini C., Mazzola S.M., Caione B., Groppetti D., Pecile A.M., Minero M., Cannas S. (2021). Influence of Gonadectomy on Canine Behavior. Animals.

[82] Moxon R., Freeman S., Payne R., Corr S.A., England W. (2022). A Prospective Cohort Study Investigating the Behavioural De-velopment of Bitches in a Guide Dog Training Programme Neutered Prepubertally or Post-Pubertally. Front. Vet. Sci..

[83] Hsu Y., Serpell J.A. (2003). Development and validation of a questionnaire for measuring behavior and temperament traits in pet dogs. J. Am.Vet. Med. Assoc..

[84] Salonen M., Mikkola S., Hakanen E., Sulkama S., Puurunen J., Lohi H. (2021). Reliability and Validity of a Dog Personality and Unwanted Behavior Survey. Animals.

[85] Turcsán B., Kubinyi E., Miklósi Á. (2011). Trainability and boldness traits differ between dog breed clusters based on conventional breed categories and genetic relatedness. Appl. Anim. Behav. Sci..

[86] Starling M.J., Branson N., Thomson P.C., McGreevy P.D. (2013). “Boldness” in the domestic dog differs among breeds and breed groups. Behav. Process..

[87] Tong W.H., Abdulai-Saiku S., Vyas A. (2019). Testosterone Reduces Fear and Causes Drastic Hypomethylation of Arginine Vaso-pressin Promoter in Medial Extended Amygdala of Male Mice. Front. Behav. Neurosci..

[88] King J.A., De Oliveira W.L., Patel N. (2005). Deficits in testosterone facilitate enhanced fear response. Psychoneuroendocrinology.

[89] Boissy A., Bouissou M.F. (1994). Effects of Androgen Treatment on Behavioral and Physiological Responses of Heifers to Fear-Eliciting Situations. Horm. Behav..

[90] Zitzmann M. (2020). Testosterone, mood, behaviour and quality of life. Andrology.

[91] Wójcik A., Powierża K. (2021). The Influence of Breed, Sex, Origin and Housing Conditions on Undesirable Behaviors in Ancient Dog Breeds. Animals.

[92] Simpson K. (2020). The Role of Testosterone in Aggression. McGill J. Med..

[93] Ayrosa F., Savalli C., Resende B. (2023). Beyond Breeding: Re-Interpreting Paradigms in Domestic Dog Aggression Research. Estud. Psicol..

[94] Duffy D.L., Hsu Y., Serpell J.A. (2020). Breed differences in canine aggression. Appl. Anim. Behav. Sci..

[95] McGreevy P.D., Georgevsky D., Carrasco J., Valenzuela M., Duffy D.L., Serpell J.A. (2013). Dog behavior co-varies with height, bodyweight and skull shape. PLoS ONE..

[96] Mariti C., Gazzano A., Moore J.L., Baragli P., Chelli L., Sighieri C. (2012). Perception of dogs’ stress by their owners. J. Vet. Behav..

[97] Olson P.N., Bowen R.A., Behrendt M.D., Olson J.D., Nett T.M. (1984). Concentrations of testosterone in canine serum during late anestrus, proestrus, estrus, and early diestrus. Am. J. Vet. Res..

[98] Hydbring-Sandberg E., Larsson E., Madej A., Hoglund O.V. (2021). Short-term effect of ovariohysterectomy on urine serotonin, cortisol, testosterone and progesterone in bitches. BMC Res. Notes.

[99] Turcu A., Smith J.M., Auchus R., Rainey W.E. (2014). Adrenal androgens and androgen precursors-definition, synthesis, regulation and physiologic actions. Compr. Physiol..

[100] Rosenfeld A.J., Lieberman J.A., Jarskog L.F. (2010). Oxytocin, Dopamine, and the Amygdala: A Neurofunctional Model of Social Cognitive Deficits in Schizophrenia. Schizoph. Bull..

[101] Kirsch P. (2005). Oxytocin Modulates Neural Circuitry for Social Cognition and Fear in Humans. J. Neurosci..

[102] Takayanagi Y., Onaka T. (2021). Roles of Oxytocin in Stress Responses, Allostasis and Resilience. Int. J. Mol. Sci..

[103] Campbell A. (2010). Oxytocin and human social behavior. Pers. Soc. Psychol. Rev..

[104] Kis A., Ciobica A., Topál J. (2017). The effect of oxytocin on human-directed social behaviour in dogs (*Canis familiaris*). Horm. Behav..

[105] Nagasawa M., Mitsui S., En S., Ohtani N., Ohta M., Sakuma Y., Onaka T., Mogi K., Kikusui T. (2015). Oxytocin-gaze positive loop and the coevolution of human-dog bonds. Science.

[106] Romero T., Nagasawa M., Mogi K., Hasegawa T., Kikusui T. (2015). Intranasal administration of oxytocin promotes social play in domestic dogs. Commun. Integr. Biol..

[107] Guvenc-Bayram G., Semen Z., Yalcin M. (2024). Investigation of the Relationship between Plasma Nesfatin-1 Levels and Neutering in Dogs. Animals.

[108] Nguyen T.V., Ducharme S., Karama S. (2017). Effects of Sex Steroids in the Human Brain. Mol. Neurobiol..

[109] Moraga-Amaro R., van Waarde A., Doorduin J., de Vries E.F.J. (2018). Sex steroid hormones and brain function: PET imaging as a tool for research. J. Neuroendocrinol..

[110] Berger M., Gray J.A., Roth B.L. (2009). The expanded biology of serotonin. Annu. Rev. Med..

[111] Nordquist N., Oreland L. (2010). Serotonin, genetic variability, behaviour, and psychiatric disorders—A review. Ups. J. Med. Sci..

[112] Seo D., Patrick C.J., Kennealy P.J. (2008). Role of Serotonin and Dopamine System Interactions in the Neurobiology of Impulsive Aggression and its Comorbidity with other Clinical Disorders. Aggress. Violent Behav..

[113] Wise R.A., Jordan C.J. (2021). Dopamine, behavior, and addiction. J. Biomed. Sci..

[114] Mârza S.M., Munteanu C., Papuc I., Radu L., Diana P., Purdoiu R.C. (2024). Behavioral, Physiological, and Pathological Approaches of Cortisol in Dogs. Animals.

[115] Rosado B., García-Belenguer S., León M., Chacón G., Villegas A., Palacio J. (2010). Blood concentrations of serotonin, cortisol and dehydroepiandrosterone in aggressive dogs. Appl. Anim. Behav. Sci..

[116] Sandri M., Colussi A., Perrotta M.G., Stefanon B. (2015). Salivary cortisol concentration in healthy dogs is affected by size, sex, and housing context. J. Vet. Behav..

[117] Frank L.A., Rohrbach B.W., Bailey E.M., West J.R., Oliver J.W. (2003). Steroid hormone concentration profiles in healthy intact and neutered dogs before and after cosyntropin administration. Domest. Anim. Endocrinol..

[118] Bencker C., Gschwandtner L., Nayman S., Grikšienė R., Nguyen B., Nater U.M., Guennoun R., Sundström-Poromaa I., Pletzer B., Bixo M. (2024). Progestagens and progesterone receptor modulation: Effects on the brain, mood, stress, and cognition in females. Front. Neuroendocrinol..

[119] Kutzler M.A. (2020). Gonad-sparing surgical sterilization in dogs. Front. Vet. Sci..

[120] Romagnoli S., Diana A., Ferré-Dolcet L., Fontaine C., Milani C. (2023). Chronic Use of Deslorelin in Dogs: Six Cases (2005–2022). Animals.

[121] Masson S., Medam T., Raibon E., Fontaine C., Levy X. (2021). Double-Blind, Placebo-Controlled Trial of Cyproterone Acetate to Prevent Flare-up Effect on Dogs Implanted with Deslorelin. Front. Vet. Sci..

